# Efficacy of a specific model for cognitive-behavioral therapy among panic disorder patients with agoraphobia: a randomized clinical trial

**DOI:** 10.1590/S1516-31802011000500008

**Published:** 2011-09-01

**Authors:** Anna Lucia Spear King, Alexandre Martins Valença, Valfrido Leão de Melo, Rafael Christophe Freire, Marco André Mezzasalma, Adriana Cardoso de Oliveira e Silva, Antonio Egidio Nardi

**Affiliations:** I MSc. Psychologist and researcher, Panic and Respiration Laboratory, Instituto de Psiquiatria da Universidade Federal do Rio de Janeiro (IPUB/UFRJ); and INCT-Translational Medicine (CNPq), Rio de Janeiro, Brazil.; II MD, PhD. Psychiatrist and researcher, Panic and Respiration Laboratory, Instituto de Psiquiatria da Universidade Federal do Rio de Janeiro (IPUB/UFRJ); and INCT-Translational Medicine (CNPq), Fluminense Federal University (UFF), Rio de Janeiro, Brazil.; III MD, MSc. Psychiatrist and researcher, Panic and Respiration Laboratory, Instituto de Psiquiatria da Universidade Federal do Rio de Janeiro (IPUB/UFRJ); and INCT-Translational Medicine (CNPq), Rio de Janeiro, Brazil.; IV PhD. Psychologist and researcher, Panic and Respiration Laboratory, Instituto de Psiquiatria da Universidade Federal do Rio de Janeiro (IPUB/UFRJ); and INCT-Translational Medicine (CNPq), Rio de Janeiro, Brazil.; V MD, PhD. Psychiatrist and coordinator, Panic and Respiration Laboratory, Instituto de Psiquiatria da Universidade Federal do Rio de Janeiro (IPUB/UFRJ); and INCT-Translational Medicine (CNPq), Rio de Janeiro, Brazil.

**Keywords:** Anxiety, Respiration, Exercise, Panic, Cognition, Ansiedade, Respiração, Exercício, Pânico, Cognição

## Abstract

**CONTEXT AND OBJECTIVE:**

Cognitive-behavioral therapy is frequently indicated for panic disorder. The aim here was to evaluate the efficacy of a model for cognitive-behavioral therapy for treating panic disorder with agoraphobia.

**DESIGN AND SETTING:**

Randomized clinical trial at Instituto de Psiquiatria da Universidade Federal do Rio de Janeiro.

**METHODS:**

A group of 50 patients with a diagnosis of panic disorder with agoraphobia was randomized into two groups to receive: a) cognitive-behavioral therapy with medication; or b) medication (tricyclic antidepressants or selective serotonin reuptake inhibitors).

**RESULTS:**

Although there was no difference between the groups after the treatment in relation to almost all variables with the exception of some items of the Sheehan disability scale and the psychosocial and environmental problems scale, the patients who received the specific therapy presented significant reductions in panic attacks, anticipatory anxiety, agoraphobia avoidance and fear of body sensations at the end of the study, in relation to the group without the therapy. On the overall functioning assessment scale, overall wellbeing increased from 60.8% to 72.5% among the patients in the group with therapy, thus differing from the group without therapy.

**CONCLUSION:**

Although both groups responded to the treatment and improved, we only observed significant differences between the interventions on some scales. The association between specific cognitive-behavioral therapy focusing on somatic complaints and pharmacological treatment was effective among this sample of patients with panic disorder and the response was similar in the group with pharmacological treatment alone.

**CLINICAL TRIAL REGISTRATION NUMBER:**

NCT 01025908

## RESUMO

**CONTEXTO E OBJETIVO:**

A terapia cognitivo-comportamental é frequentemente indicada para o transtorno de pânico. O objetivo foi avaliar a eficácia de um modelo de terapia cognitivo-comportamental no tratamento do transtorno de pânico com agorafobia.

**TIPO DE ESTUDO E LOCAL:**

Ensaio clínico aleatório no Instituto de Psiquiatria da Universidade Federal do Rio de Janeiro.

**MÉTODOS:**

Um grupo de 50 pacientes com diagnóstico de transtorno de pânico com agorafobia foi randomizado em dois grupos para receber a) terapia cognitivo-comportamental com medicação ou b) medicação (antidepressivos tricíclicos ou inibidores seletivos da recaptação da serotonina) sem terapia.

**RESULTADOS:**

Embora não se tenha observado diferença entre os grupos após o tratamento em quase todas as variáveis, com exceção de alguns itens da Escala de Incapacitação de Sheehan e da Escala de Problemas Psicossociais e Ambientais, ao final do estudo os pacientes que receberam a terapia específica apresentaram significativa redução dos ataques de pânico, ansiedade antecipatória, esquiva agorafóbica e medo das sensações corporais em relação ao grupo sem a terapia. Na escala de avaliação global do funcionamento aumentou o bem-estar global de 60,8% para 72,5% entre os pacientes do primeiro grupo, diferentemente do segundo grupo.

**CONCLUSÃO:**

Embora ambos os grupos tenham respondido ao tratamento e melhorado, observamos diferença significativa entre as duas intervenções apenas em poucas escalas. A associação de terapia cognitivo-comportamental específica centrada nas queixas corporais, associada ao tratamento farmacológico, foi eficaz nesta amostra de pacientes com transtorno de pânico e agorafobia e semelhante ao grupo apenas com tratamento farmacológico.

## INTRODUCTION

Panic disorder is characterized by frequent and recurring acute anxiety attacks, which result in inappropriate phobic behavior.^1^ Previously, panic disorder was seen as an occurrence without triggering factors. Today, a relationship between panic disorder and fear of specific triggering body sensations has been shown.^2^

According to cognitive models,^3^ panic attacks can begin with stressful events and with thoughts or situations that are perceived as such, even though there is no real danger. Panic disorder is usually said to be present from the time when anxious individuals begin to wrongly associate body sensations such as palpitations, sweating, feelings of shortness of breath, dizziness and loss of control, among others, with a serious disease or imminent death. The natural adaptive physiological reactions deriving from the biological mechanism of "fight or flight"^4-21^ become mixed up with symptoms similar to those resulting from severe situations.

The catastrophic cognition thus established becomes individuals’ effective behavioral pattern. Therefore, external activities that produce body sensations similar to the sensations experienced during panic attacks, including physical exercise,^5^ sexual stimuli, caffeine intake and thermal changes, among others, normally trigger panic attacks^2^ in panic disorder patients.

Cognitive-behavioral therapy^6^ (CBT) is highly indicated for cases of panic disorder since it is a rapid therapeutic method with techniques that neutralize and/or reorganize maladaptive behaviors resulting from this disorder. In CBT, patients are told to describe their emotions in physiological, cognitive and behavioral terms, in an attempt to identify the compromised cognition that has resulted in distorted responses. It also attempts to develop cognitive reorganization conduct, in order to restore real values^7^ for their mistaken beliefs.

With CBT, symptoms similar to those of panic attacks are introduced, in a reliable manner, through specific physical exercises, with the purpose of allowing such individuals to perceive that there is no reason to fear their own sensations. The objective is to make patients understand the origin of their body sensations, learn to cope with them and demystify the interpretation that they are dangerous.

These symptom induction exercises^2^ cause hyperventilation, sweating, dizziness and palpitation, among others. They are performed in a safe environment, in which patients perceive, without limiting anxiety, that the emerging body sensations are harmless and are the result of physiological adaptation mechanisms. Through understanding these issues, individuals are able to recover the cause of their distorted cognition, which helps them develop new strategies to deal with their thoughts, physical feelings and behavior.

Cognitive reorganization^8^ is another key factor in CBT treatment, since it helps patients to identify, change and question established patterns, thereby giving new meaning to maladaptive cognitions that sustain the fear in certain situations.

As soon as panic disorder patients understand the personal negative cognitions that precede emotional and reorganized reactions, they are ready to try "*in vivo*" exposure.^8^ In this procedure, individuals are confronted with feared or avoided situations, within a personal hierarchy that they create, experience and rate on a scale from least stressful to most feared. Repeated confrontation with each item of the hierarchy enables development of alternatives differing from those that are normally used. Patients learn that they can control and deal with physical sensations, and learn that the expected catastrophic consequences do not take place.

The present study was based on methods and results from previous studies^2^ and it attempted to achieve the best way of combining panic attack and agoraphobia control treatments using CBT, with pharmacological treatment. This study proposed a program of 16 one-hour individual CBT sessions, in which patients’ issues were addressed. There was also a standard learning content in all of the CBT sessions, aimed at making it easier for patients to cope with panic disorder and understand its origin, nature and path.^6^

## OBJECTIVE

The purpose of this study was to assess the efficacy of the proposed CBT model for treating panic disorder patients with agoraphobia, which could become an ally of pharmacological treatment. We hypothesized that the group of patients receiving the medication interventions and CBT (intervention group) would show significant changes in their behavior, such as remission or reduction of anxiety, panic attacks, anticipatory anxiety, fear of body sensations, loss of control and agoraphobia avoidance. Furthermore, we hypothesized that they would present a significant change in the general evaluation of well-being, between the beginning and end of the treatment, in comparison with the control group (medication without CBT), over the same period.

## MATERIALS AND METHODS

This randomized clinical trial (RCT) used validated instruments to assess a rapid specific CBT model among a sample of 50 volunteer patients with panic disorder and agoraphobia who were being treated at Instituto de Psiquiatria da Universidade Federal do Rio de Janeiro, in its Panic and Respiration Laboratory. The patients were referred by the psychiatric physicians in the team. The diagnosis of panic disorder was made in accordance with the diagnostic criteria in the Diagnostic and Statistical Manual of Mental Disorders^1^ and the Structured Clinical Interview diagnostic assessment instrument.^9^ The medical procedure consisted of prescribing tricyclic antidepressants or selective serotonin reuptake inhibitors.

The inclusion criteria for the study were that the patients should be over 18 years old of either gender with a diagnosis of panic disorder and agoraphobia, but without severe comorbidities such as bipolar disorder, schizophrenia and other psychotic disorders, mental retardation, mental disorder due to a general medical condition or alcohol and substance-related disorders.

The patients were informed about the study and assured that they could withdraw at any moment, without losing their medical and psychological benefits. If they agreed to participate, they signed an informed consent statement and were made aware of all the procedures to be adopted. They were also assured that the data and results obtained in the study would be kept confidential in accordance with the ethical principles for medical research involving human subjects, in force at Instituto de Psiquiatria da Universidade Federal do Rio de Janeiro. The project was approved by the Research Ethics Committee, in compliance with the Helsinki Declaration.

The sample was divided into two groups. The first group, named the intervention group, was composed of 25 individuals who received 16 CBT sessions along with medication. The second group, named the control group, was also composed of 25 individuals. These patients only received medication, without concomitant CBT. The patients in both groups were randomly selected. The randomization was done by means of sealed envelopes and was performed by a researcher not directly involved in patient evaluations. The evaluators did not have access to the envelopes during the study, and thus did not know which patients were receiving CBT and which were not. During the CBT sessions, the topic of medications was not mentioned to the patients at any time. However, they could talk about medications during their visits to their physicians. The physician who prescribed the drugs did not participate in implementing the instruments relating to the study. Patients did not talk to the evaluator about the treatment that they were receiving.

The 16 CBT sessions were based on previous studies,^2^ with changes and adaptations according to the characteristics of the sample of patients treated. The sessions included the following topics: clarification of the course of panic disorder by explaining the concepts of anxiety, agoraphobia, panic, hyperventilation, breathing retraining exercise and muscle relaxation; development of hierarchies for "patients’ fears" from the least to the most stressful; identification of maladaptive cognition and cognitive reorganization; symptom induction exercises; interoceptive exposure; introduction to "*in vivo*" exposure; strengthening of achievements; observation of difficulties in the procedure; and maintenance of treatment gains.

### Standard model for the 16 CBT sessions

Session 1: The course of panic disorder was explained: the concepts of anxiety, fear, panic, and agoraphobia. A hierarchy for feared situations was developed.

Session 2: The physiological "fight and flight" mechanism, and similarities between the sensations resulting from this biological mechanism and the body sensations emerging from panic were explored. We explained how to perform the breathing retraining exercise used in the sessions: the patients were asked to put their hands on their stomach to feel the air passing through the diaphragm, and the abdomen expanding and contracting with each breath taken. The patients were instructed to inhale slowly through the nose and count to three, while holding their breath, and then to exhale the air slowly through the mouth, while counting to six. The breathing exercise was then repeated a few more times.

Session 3: The hyperventilation model was defined as the rhythm and depth of breathing that was exaggerated in relation to the body's needs at a specific moment. The hyperventilation symptom induction exercise (SIE) was performed while patients were seated: they inhaled and exhaled deeply for 90 seconds, and then, a breath holding exercise (BRE) was performed while the patients were standing up and staring at a light spot on the ceiling.

Session 4: While seated, the patients stared at the light for one minute and then tried to read. We analyzed the symptoms and patterns of negative and catastrophic repetitive thoughts that appeared right after the symptoms occurred. The patients understood the origin of the symptoms and perceived them as harmless. Breathing retraining exercises were then performed. The patients were always instructed to describe their emotions in physiological, cognitive and behavioral terms.

Session 5: Body sensation concepts that could be intensified according to the situation or substance were explored. These included exercising and moving abruptly, temperature changes, bright lights, caffeine, alcohol, medications and irregular breathing patterns. The panic was grounded in the fear of physical sensations. We performed a SIE with the patient turning around in one place for one minute and then stopping to understand the reactions. Then breathing retraining exercises were performed.

Session 6: Some of the complaints of the panic disorder patients were: "I can't breathe," or "I'm suffocating." We explained that it was natural to breathe heavily when they were nervous. In such situations, the body seeks more energy in the form of oxygen to prepare itself to cope with danger. When oxygen is not used in the same proportions as consumed, hyperventilation occurs. We went back to the hyperventilation concepts and redid the SIE in Session 4 followed by breathing retraining exercises.

Session 7: The patients learned about cognitive reorganization. The main point was to understand that the interpretation of the events determined the nature of the resulting emotional reactions. In this session, we tried to help the patients to identify and give new meaning to many specific maladaptive cognitions. The anticipatory anxiety and agoraphobia avoidance concepts^4^ were discussed and clarified.

We also investigated the myths that patients held in relation to panic attacks: "I think I'm losing control," "I think I'm going to die," "I think I'm having a heart attack," "I think I'm going crazy," among others. The patients were instructed to question and take an objective view of their assumptions and beliefs, concentrate on the realistic probabilities and gather evidence and ways of dealing with the situations. In this session, we looked at any myths that the patients might have, identified the distorted ideas, and tried to give them a new meaning.

Session 8: Interoceptive exposure,^8^ referring to the learned fear of internal states, was dealt with. Certain sensations of horror, similar to fears previously experienced, would tend to trigger the possibility of a new panic attack. Two symptom induction exercises were performed and the symptoms were analyzed in an attempt to understand their causes. In the first exercise, the patients were asked to sit down, put their head between their legs for 30 seconds and then lift their head up and look at the ceiling. In the second exercise, they were asked to hold their breath for as long as they could while turning around in one place (30 seconds). Breathing retraining exercises were then performed.

Session 9: Three symptom induction exercises were performed, The exercise in Session 5 and the two exercises in Session 8 were undertaken, followed by breathing retraining exercises. Previously, the patients had a distorted association of the facts, interpreting palpitation, sweating and shortness of breath, among others, as signs of imminent death or loss of control. After performing the symptom induction exercises, the patients began to realize that the sensations produced, which were similar to panic, were harmless: caused by specific stimuli, without producing the feared consequences.

Session 10: Symptom induction exercises were performed again. While standing, the patients made head movements from side to side for 30 seconds and then stopped and tried to stare at a spot on the wall. Next, we evaluated the resulting body sensations and tried to understand the cause. We helped the patients to recover their physical and respiratory balance through breathing retraining exercises. We also introduced the "*in vivo*" concept, in which the patients confronted agoraphobic situations. We explained that their agoraphobia was sustained by the fear of panicking or experiencing certain body sensations. Hence, learning in the first phase of the treatment that body sensations relating to panic were not actually dangerous was essential for overcoming agoraphobia and controlling the panic. The real confrontation with the feared situation was repeated with each item in the patients’ fear hierarchy, beginning with the least anxiety-producing item and ending with the most feared situation. We asked the patients to perform "*in vivo*" exposure whenever they had the chance.

Session 11: We investigated the symptom induction procedures performed by the patients alone and observed their behavior. We reinforced the concept that the panic cycle began with anticipation of a feared situation, i.e. "What you choose to think is what you choose to feel." By avoiding confronting the feared situation, patients’ understanding that they are unable to fight it is reinforced. On the other hand, by confronting it and winning, patients not only reinforce their courage but also come to understand that they are capable of fighting.

Session 12: A muscle relaxation technique was performed, and repeated whenever necessary. The patients were told to close their eyes and take a deep breath using the diaphragm at their own pace, to relax. Then, while slowly inhaling and exhaling, they were instructed to apply tension to four muscle groups of the body by contracting them as much as possible for ten seconds and then relaxing for another 15 or 20 seconds. The muscle groups required in this exercise were: 1- Face; 2- Arms, shoulders, chest and neck; 3- Abdomen, backbone and genital organs; 4- Legs and feet.

Session 13: Treatment for anticipatory anxiety was provided. The patients needed to understand that when they thought beforehand of a feared situation, the brain should understand that it was approaching an emergency, i.e. a dangerous situation. Their memories usually recreated sensations similar to those of old traumas. Through succeeding in changing the thoughts relating to the capacity to cope with feared situations, physical symptoms can then be controlled. Even in safe situations, if the mind perceives them as unsafe, the body will react to them as a message of danger, thereby producing symptoms.

Session 14: The patients were encouraged to use cognitive strategies such as repeated exposure to changes in temperature, and physical exercises at home, with the purpose of inducing and tolerating the sensations (without the therapist's help). This allowed them to achieve their own individual cognitive reorganization and lose the fear of being alone with their own body sensation. It also allowed them to perceive that their physiological reactions were not normal, could be coped with and did not result from a poorly made association in which they believed that the symptoms resulted from something serious.

Session 15: We analyzed, identified and questioned the thoughts experienced in agoraphobic situations by the patients during their attacks, and helped them with their dysfunctional thoughts and cognitive reorganization. We indicated and tried to correct the maladaptive coping methods used by the patients, such as the "need" for someone else as a sign of safety and the "need" for objects such as telephones, money and medication. We told the patients that the safety signals resulted in sustaining their mistaken belief of danger.

Session 16: We reviewed the "*in vivo*" exposure that had been performed, encouraged repetitions and discussed the difficulties of the procedures. We reminded the patients that running away from "*in vivo*" exposure reinforced and sustained fear. We encouraged the patients to think about the voided tasks and helped them with cognitive reorganization. We mentioned the fact that additional diagnoses tend to decrease after panic disorder has been treated. Such comorbidities could include depression, generalized anxiety and social phobia. We told the patients that the medication should work as an aid and not create dependency, which would deprive individual of the ability to learning from the evident emotional "material." Thus, contact with emotions would be necessary in order to learn to cope with them. In this final session, an overall review of the main CBT concepts used during the treatment was made.

After all the research procedures had been completed, within the same period of time, we reassessed both groups using the same instruments as used initially. We compared the groups and observed the changes, benefits, losses and resulting differences between the group that received CBT with medication and the control group, which received medication alone, without CBT.

With the purpose of comparing the results from the two groups after the interventions, the following assessment instruments were used at the beginning and end of the study: the Beck anxiety inventory (BAI);^10^ the state-trait anxiety inventory (STAI);^11^ the Sheehan disability scale;^12^ the psychosocial and environmental problems (PEP) scale (Axis IV);^1^ the overall functioning assessment scale (Axis V);^1^ the fear and phobia questionnaire (FPQ);^13^ the agoraphobia cognitions questionnaire (ACQ);^14^ the panic and agoraphobia scale (PAS)^15^ and the mobility inventory.^16^

It was deemed that a response had been achieved from the intervention if the following reductions in scores from the baseline were observed: 50% in the BAI, 30% in the STAI, 50% in the Sheehan disability scale (total), 50% in the FPQ (total), 50% in the ACQ (total), 40% in the body sensation questionnaire, 50% in the PAS (total), 30% in the mobility inventory (total), 30% in the general wellbeing assessment and 30% in the PEP scale (total).

### Statistical analysis

The test results were characterized according to the patients’ sociodemographic data, psychiatric and physical comorbidities and use of medication (such as tricyclic antidepressants and selective serotonin reuptake inhibitors).

During the assessment, the chi-square (χ^2^) or analysis of variance (ANOVA) test was performed, depending on the variable measured, with 95% confidence intervals or *p*-values ≤ 0.05. In this, the tests were performed using the values found at the beginning of the treatment.

Analyses measuring any differences between the beginning and end of the treatment were performed, thereby making it possible to determine the efficacy of the actions taken. The purpose was to identify statistically significant differences in the final results from the tests.

In the analysis on the results (below), the group of patients with CBT and medication was referred to as the "intervention" group and the group of patients without CBT but with medication, as the "control" group.

For the CBT group to present a statistically significant difference of 5%, given a minimum difference of 50% between the groups in the total score from the Beck Anxiety Inventory, it was necessary to have 25 patients in each group for a confidence interval of 95% (α = 0.05) and a statistical power of 80% (β = 0.02).

## RESULTS

### 1. Description of the Groups:

#### Characterization of the total sample of the study (n = 50)

Gender: 78% were female patients and 22% male patients.

Marital status: 42% were married, 36% single and 22% separated/divorced.

Level of education: 28% had graduated from middle school, 38% from high school and 34% from university (bachelor or postgraduate level).

Clinical comorbidities: 42% did not show any clinical comorbidities and 58% showed some type of clinical comorbidity.

Smoking: 44% were nonsmokers, 14% were smokers and 42% had quit smoking.

Respiratory subtype: 70% presented the respiratory subtype of panic disorder.

#### Participants’ ages

The intervention and control groups differed significantly in age. The minimum age for both groups was 22 and the maximum was more than 55. However, the mean age for the intervention group was 44.5 (standard deviation, SD = 12.8; 95% confidence interval, CI = 39.0-49.8) and for the control group, it was 33.7 (SD = 9.6; 95% CI = 29.7-37.7). The ANOVA test on the means showed significance of 0.000, i.e. lower than the *p*-value of 0.05, with 95% confidence.

#### Age at onset of panic disorder

There were also significant differences in age at the onset of panic disorder. In the control group (which was a younger group), the disorder was identified earlier than among the patients in the intervention group (which was an older group) with CBT. Among the controls, the mean age at onset was 27.1 (SD = 6.8; 95% CI = 24.3-29.9), whereas it was 33.5 (SD = 11.0; 95% CI = 29.0-38.1) in the intervention group. The ANOVA test on the means showed significance of 0.017, which was lower than the p-value of 0.05, with 95% confidence. Table 1 shows the age differences at the onset of panic disorder, within the age groups.

**Table 1:** Age groups at the onset of panic disorder at the intervention and control groups

Age groups at the onset of panic disorder	Control group(%)	Intervention group (%)
18-29 years	64	32
30-39 years	32	32
40 years or over	3	36

The results suggested that there was a strong correlation between the age of the individuals undergoing treatment and the age at which panic disorder was detected. Individuals undergoing treatment at a greater mean age, like those in the intervention group, either had a late onset of the disorder or a late diagnosis of the disorder. The contrary was observed in the control group.

There was a correlation in both groups between the age of the individuals undergoing treatment and the age at which they began to experience panic disorder. In the control group, the correlation was stronger than in the intervention group. Among the controls, 57% of the variation in age at onset was explained only by the individuals’ ages; while in the intervention group, 37% of the variation in age at onset was explained by the individuals’ ages.

Although this correlation was stronger in the control group, in both cases the mean difference between the age at onset and the current age was eight years.

#### Individual and family income

Although no differences were found between the groups in relation to the individuals’ education levels, there was a clear difference in relation to individual and family income levels. Among the patients in the intervention group, individual income could be twice as much as the income in the control group. In relation to family income, the difference remained, although somewhat smaller (1.6 times).

These differences were closely related to the age difference between the groups, since incomes tended to be higher at older ages. Pearson's correlation between age and individual income was 0.401 with 99% confidence (intervention group) and 0.417 with 99% confidence (control group).

#### Psychiatric comorbidities:

The psychiatric comorbidity investigated was depression. The two groups differed in relation to depression. Among the patients in the intervention group, 28% showed this type of comorbidity, versus 68% in the control group. This meant that the control group had more than twice as many cases of depression episodes as seen in the intervention group. To come to this conclusion, a chi-square test was performed, with 95% confidence (P-value ≤ 0.05). In most cases, depression was mild. Table 2 shows the depression episodes in the intervention and control groups.

**Table 2:** Percentage of previous depressive episodes in the intervention and control groups

Control group (%)	Intervention group (%)	
32	72	Absent
68	28	Previous

### 2. Medication:

Table 3 shows the percentages of group members using medications at the beginning of the treatment.

**Table 3:** Percentage of group subjects using medication at the beginning of the intervention

Medication	Intervention group (%)	Control group (%)
Current use of tricyclic antidepressants	44	64
Current use of selective serotonin reuptake inhibitors	56	28
Current use of benzodiazepine	0	0
Current use of other antidepressants	0	0
Use of anticonvulsants	0	0
Use of antipsychotics	0	0

### 3. Assessment scales

The two groups had similar baseline scores for all the scales (Table 4).

**Table 4:** Baseline mean scores in intervention group and control group according to ANOVA*

Tests	Intervention group	Control group	Significant difference†
BIA (Beck anxiety inventory)	34.9	33.6	0.756
STAI-S (State and Trait Anxiety Inventory part 1)	51.7	50.4	0.563
STAI-T (anxiety inventory part 2)	54.8	52.9	0.654
Sheehan disability scale – total	14.6	15.8	0.788
Sheehan – work	5.2	5.5	0.538
Sheehan – social	4.8	4.4	0.741
Sheehan – family	4.6	4.9	0.648
Fear and phobia questionnaire (FPQ) – total	49.4	50.7	0.833
FPQ – agoraphobia score	20.2	21.2	0.398
FPQ – blood score	15.6	14.8	0.566
FPQ – social score	13.7	13.4	0.749
Agoraphobia cognition questionnaire (ACQ)	2.7	2.6	0.692
ACQ – loss of control	2.8	2.7	0.891
ACQ – physical problems	2.6	2.3	0.740
Body sensations questionnaire	3.1	3.7	0.732
Panic and agoraphobia scale (PAS)	27.9	28.2	0.505
PAS – panic attacks	1.4	1.3	0.673
PAS – agoraphobia avoidance	2.4	2.5	0.828
PAS – anticipatory anxiety	2.9	3.1	0.442
PAS – disability	2.2	2.1	0.807
PAS – precautions	2.1	2.4	0.467
Mobility inventory – accompanied	2.4	2.3	0.543
Mobility inventory – alone	3.5	3.7	0.604
General wellbeing assessment	60.8	61.5	0.921
Psychosocial and environmental problems (PEP)	72.0	71.6	0.840
PEP – social	56.0	55.8	0.849
PEP – education	4.0	3.8	0.783
PEP – occupational	68.0	67.0	0.589
PEP – housing	32.0	33.0	0.825
PEP – economic	60.0	59.0	0.410
PEP – access to healthcare	20.0	20.0	0.359
PEP – legal problems‡	0.0	0.0	–
PEP – other	12.0	11.0	0.784

* Analysis of variance. The PEP results are percentages of those showing any PEP (chi-square);

† Significant differences with 95% confidence, or P-value ≤ 0.05;

‡ None of the intervention group members showed any PEP legal problems, and therefore the chi-square test was not performed.

#### Intervention group at the beginning and end of CBT

There were significant differences in the scores for the intervention group between the baseline and the end of the treatment (Table 5). Among the nine tests performed, six (67%) showed positive results. For example, the panic and agoraphobia scale showed satisfactory results regarding decreases in the symptoms of anticipatory anxiety from 2.9% to 2.1%; panic attacks from 1.4% to 0.7%; and agoraphobia avoidance from 2.4% to 1.6%. The general wellbeing scale (with scores from 0 to 100) showed a significant increase in the wellbeing of the individuals in the intervention group, from 60.8% to 72.5%, after the treatment with CBT (Table 5).

**Table 5:** Intervention group scores at the beginning and end of the trial according to ANOVA*

Tests	Intervention group		Significant difference†
	Beginning of CBT	End of CBT	
BIA (Beck anxiety inventory)	34.9	20.0	0.001
STAI-S (State and Trait anxiety inventory part 1)	51.7	43.9	0.053
STAI-T (State and Trait anxiety inventory part 2)	54.8	50.4	0.274
Sheehan disability scale – total	14.6	4.4	0.000
Sheehan – work	5.2	0.4	0.000
Sheehan – social	4.8	2.2	0.009
Sheehan – family	4.6	1.8	0.005
Fear and phobia questionnaire (FPQ) – total	49.4	38.3	0.186
FPQ – agoraphobia score	20.2	14.7	0.132
FPQ – blood score	15.6	11.6	0.207
FPQ – social score	13.7	12.0	0.584
Agoraphobia cognition questionnaire (ACQ)	2.7	2.1	0.012
ACQ – loss of control	2.8	2.1	0.021
ACQ – physical problems	2.6	2.0	0.027
Body sensations questionnaire	3.1	2.4	0.008
Panic and agoraphobia scale (PAS)	27.9	18.6	0.012
PAS – panic attacks	1.4	0.7	0.016
PAS – agoraphobia avoidance	2.4	1.6	0.033
PAS – anticipatory anxiety	2.9	2.1	0.028
PAS – disability	2.2	1.6	0.109
PAS – precautions	2.1	1.5	0.117
Mobility inventory – accompanied	2.4	1.9	0.056
Mobility inventory – alone	3.5	3.0	0.183
General wellbeing assessment	60.8	72.5	0.015
Psychosocial and environmental problems (PEP)	72.0	44.0	0.045
PEP – social	56.0	28.0	0.045
PEP – education	4.0	4.0	1.000
PEP – occupational	68.0	52.0	0.248
PEP – housing	32.0	24.0	0.529
PEP – economic	60.0	56.0	0.774
PEP – access to healthcare	20.0	0.0	0.018
PEP – legal problems‡	0.0	0.0	–
PEP – other	12.0	8.0	0.637

* Analysis of variance. The PEP results are percentages of those showing any PEP (chi-square);

† Significant differences with 95% confidence, or P-value ≤ 0.05;

‡ None of the intervention group members showed any PEP legal problems, and therefore the chi-square test was not performed.

Additionally, taking into consideration all the tests and their subitems, out of the 33 tests performed, 17 (51%) showed significant differences.

Comparing the initial evaluations on the intervention group with the final evaluations using the same instruments after 16 CBT sessions, significant differences were observed in relation to the Beck anxiety inventory,^10^ showing a general reduction in anxiety from 34.9% to 20.0%, and in relation to the Sheehan disability scale,^12^ with a reductions in individuals’ disability at work from 5.2% to 0.4%, in their social lives from 4.8% to 22% and in their family lives from 4.6% to 1.8%. This shows that the overall feeling of personal disability in the intervention group dropped from 14.6% to 4.4%.

In the agoraphobic cognition questionnaire,^14^ the intervention group showed a reduction in the loss of control from 2.8% to 2.1%, and a reduction in the fear of physical problems from 2.6% to 2.0%.

Another important factor was observed through the body sensations questionnaire,^14^ in which some of the main symptoms resulting from panic disorder, such as palpitation, shortness of breath, dizziness, tingling, nausea, sweating and disorientation, among others, were evaluated. The results showed a general reduction from 3.1% to 2.4% in the intervention group, in all respects.

The panic and agoraphobia scale^15^ showed reductions in panic attacks from 1.4% to 0.7%, agoraphobia avoidance from 2.4% to 1.6% and anticipatory anxiety from 2.9% to 2.1%. These results reveal that, in a general manner, the intervention group was able to reduce some of the main behavioral patterns evident in panic disorder, from 27.9% to 18.6%.

The psychosocial and environmental problems scale^1^ showed that there were reductions in the problems relating to the social environmental from 56.0% to 28.0% and in the problems relating to access to healthcare services from 20.0% to 0.0%.

According to the overall functioning assessment scale,^1^ which evaluated individuals’ psychological, social and occupational functioning within a hypothetical mental health-disease continuum, the intervention group showed a statistically significant increase in overall wellbeing from 60.8% to 72.5%.

No statistically significant difference was shown in the control group, in the tests performed to compare the beginning and end of the study.

The patients who underwent CBT (intervention group) showed statistically significant differences in relation to the control group in two of the nine tests performed (22%): the Sheehan disability scale (total, work and family) and the psychosocial and environmental problems scale (access to healthcare). Among all the types of test and their subitems, 15% (5 out of 33) showed some kind of important variation. The control group did not show any statistically significant difference, unlike the intervention group.

The results from the Sheehan Scale showed that the only factor relevant to understanding the difference between the total Sheehan and work Sheehan values was their close association with individuals presenting psychiatric comorbidities, after controlling for other variables. It should be noted that the control group included more individuals with psychiatric comorbidities than did the intervention group. This factor may explain the distinction between the Sheehan total and Sheehan work test results. The psychiatric comorbidity of depression was responsible for depression symptoms that may have interfered with these individuals’ work and social lives.

The variation in the Sheehan family scale was significant in relation to the final means for the intervention and control groups, but ceased to be relevant after the result was controlled for other variables. This meant that if any differences between the intervention and control groups actually existed, they were probably related to the treatment, with or without CBT.

The psychosocial environmental problems test (Table 5) showed relevant results for the intervention group. In this case, CBT was the explanatory variable capable of increasing or decreasing the chance that individuals would have psychosocial environmental problems. Those who did not undergo CBT had a 400% higher chance of developing psychosocial environmental problems than did those who underwent CBT.

Table 6 presents a comparison of the results (percentage of responders) between the intervention and control groups. We observed that although both groups responded, there was no difference between the groups at the end of treatment in relation to almost all the variables, with the exception of some items of the Sheehan disability scale and the psychosocial and environmental problems scale. Significant differences were observed only in a few items of two general scales.

**Table 6:** Comparison between the intervention and control groups: percentage of responders at the end of the treatment period

	Intervention group n = 25	Control group n = 25	Significant difference*
	%	%	
BIA (Beck anxiety inventory)	60	56	0.116
STAI-S (State and Trait anxiety inventory part 1)	52	44	0.395
STAI-T (State and Trait anxiety inventory part 2)	40	36	0.213
Sheehan disability scale - total	44	36	0.013
Sheehan – work	52	60	0.001
Sheehan – social	32	28	0.179
Sheehan – family	48	44	0.035
Fear and phobia questionnaire (FPQ) – total	56	60	0.872
FPQ – agoraphobia score	64	68	0.860
FPQ – blood score	48	40	0.431
FPQ – social score	52	60	0.882
Agoraphobic cognition questionnaire (ACQ)	60	52	0.233
ACQ – loss of control	68	76	0.384
ACQ – physical problems	44	40	0.161
Body sensations questionnaire	56	52	0.957
Panic agoraphobia scale (PAS)	64	70	0.304
PAS – panic attacks	72	64	0.149
PAS – agoraphobia avoidance	56	60	0.965
PAS – anticipatory anxiety	52	48	0.609
PAS – disability	56	52	0.359
PAS – precautions	60	68	0.098
Mobility inventory – accompanied	40	36	0.235
Mobility inventory – alone	52	48	0.869
General wellbeing assessment	68	60	0.769
Psychosocial and environmental problems (PEP)	72	68	0.009
PEP – social	68	64	1.000
PEP – education	40	44	1.000
PEP – occupational	76	72	0.395
PEP – housing	68	72	0.355
PEP – economic	64	60	1.000
PEP - access to healthcare	20	24	0.018
PEP – other	28	32	0.552

* Significant differences with 95% confidence, or P-value ≤ 0.050.

#### Analysis on the respiratory subtype

Table 7 shows the analysis on the respiratory subtype^17^ in the intervention and control groups.

**Table 7:** Analysis on the respiratory subtype. The results showed that 77.6% of the sample of panic disorder patients from both groups presented the respiratory subtype^20^ while 22.4% presented the non-respiratory subtype

Panic disorder subtype	Final assessment		Total
	Intervention	Control	
Respiratory	75.0	80.0	77.6
Non-respiratory	25.0	20.0	22.4
Total	100.0	100.0	100.0
Chi-square = 0.675			

## DISCUSSION

The learning content of the 16 CBT sessions helped the patients to cope with and understand the nature of panic attacks and how they are triggered.

Lack of panic attack prediction and control increases general anxiety over the events that emerge and contributes towards high levels of chronic anxiety.^4^ Severe personal, medical, financial and social consequences arise when anxiety disorders are not treated. Moreover, they can also be associated with other psychiatric disorders, such as depression, substance abuse and high suicide potential.^18^

According to Barsky et al.,^19^ patients with panic disorder can develop hypochondria, among other conditions. This is a disorder characterized by concern or fear of acquiring a severe disease, as a consequence of distorted cognition that associates body sensations with something dangerous.

The results showed that in relation to agoraphobia cognition, the frequency of negative thoughts or ideas of hypocondria^20^ that emerged when the patients were nervous or frightened was reduced from 2.7% to 2.1% among the patients in the intervention group.

The intervention group (with CBT) and the control group (without CBT) differed significantly in age. The minimum age of both groups was 22 years and the maximum age was 55 years or over. However, the mean age in the intervention group was 44.6 (± 12.7) years, while it was 33.7 (± 9.6) years in the control group. Whereas 80% of the control group was composed of individuals aged 39 years and younger, only 32% fell into this age group in the intervention group. Although unintentionally, this difference may have interfered with the results because of the strong correlation between the age of the patients undergoing treated and the age at which the disorder was first detected. It is possible that delays in seeking treatment contributed towards increasing the severity of agoraphobia and comorbid depression.

Individuals undergoing treatment at a higher mean age, such as those in the intervention group with CBT, had either a late onset of panic disorder or a late diagnosis of the disorder, unlike the control group. There were also significant age variations regarding the onset of panic disorder. In the control group (which was a younger group), the disorder was detected earlier than in the intervention group (which was an older group). Among the controls, the mean age was 27.1 (± 6.7), while it was 33.4 (± 10.9) in the intervention group.

One limitation of this study was that there was no stratified randomization to make the two groups more similar. This may have constituted a source of bias. We certainly recognize that the differences between the intervention and control groups (age, comorbidities etc.) may have contributed towards the outcome of the study. However, even taking this point into consideration, the group that received medication and CBT showed significant improvements in the scores, between the baseline and the end of the treatment, unlike the control group. This shows the importance of CBT in association with medications for treating panic disorder patients. It has been shown^2^ that the results produced by combining CBT with pharmacotherapy are better than those from pharmacotherapy alone. Another limitation observed was that the groups differed in relation to the presence of the psychiatric comorbidity of mild depression. Among the patients in the intervention group, 72% lacked this type of comorbidity, compared with only 32% in the control group. This means that the control group had more than twice as many cases of depression as did the intervention group with CBT. Medication-oriented studies that adequately assess the results and extent of the improvement among panic disorder patients are required.

The literature reveals that depression is one of the most frequently found comorbidities in psychiatric disorders.^1^ The present study investigated the presence of clinical and psychiatric comorbidities, such as depression, and observed the behavior of this comorbidity by means of scales, during the treatment.

The patients’ achievements consisted of reinforcement of new and safer responses to phobic situations. The panic disorder patients treated with medication alone showed slower recovery than did those whose treatments were combined with CBT. A larger number of patients enrolled for and collaborated with CBT.

We fulfilled the purpose of the study, which was to test and observe the efficacy and limitations of the proposed CBT model for treating panic disorder patients. There is a need to verify whether the results and differences found at the end of the treatment were actually due to CBT, or whether they were due to the variations in group profile that existed, such as age, presence of psychiatric comorbidities and differential use of drugs (such as tricyclic antidepressants and selective serotonin reuptake inhibitors). It is also necessary to have more control over the study sample and variables, in order to evaluate more precisely the effect of CBT and other treatments for panic disorder.

## CONCLUSION

The association between specific cognitive-behavioral therapy focusing on somatic complaints and pharmacological treatment was effective. The response was similar to pharmacological treatment alone among panic disorder patients with agoraphobia. We were not able to prove our hypothesis that the intervention group with CBT would show significant changes in their clinical parameters, from the baseline to the end of treatment, with a difference in relation to the control group. This was observed only for a few items of two general scales.
